# Membrane Technology for the Recovery of Lignin: A Review

**DOI:** 10.3390/membranes6030042

**Published:** 2016-09-06

**Authors:** Daniel Humpert, Mehrdad Ebrahimi, Peter Czermak

**Affiliations:** 1 Institute of Bioprocess Engineering and Pharmaceutical Technology, University of Applied Sciences Mittelhessen, Giessen 35390, Germany; daniel.humpert@lse.thm.de (D.H.); mehrdad.ebrahimi@lse.thm.de (M.E.); 2 Department of Chemical Engineering, Kansas State University, Manhattan, KS 66506, USA; 3 Faculty of Biology and Chemistry, Justus-Liebig University of Giessen, Giessen 35390, Germany

**Keywords:** ceramic membrane, lignin treatment, lignin fractionation, lignosulfonate, spent sulfite liquor

## Abstract

Utilization of renewable resources is becoming increasingly important, and only sustainable processes that convert such resources into useful products can achieve environmentally beneficial economic growth. Wastewater from the pulp and paper industry is an unutilized resource offering the potential to recover valuable products such as lignin, pigments, and water [1]. The recovery of lignin is particularly important because it has many applications, and membrane technology has been investigated as the basis of innovative recovery solutions. The concentration of lignin can be increased from 62 to 285 g∙L^−1^ using membranes and the recovered lignin is extremely pure. Membrane technology is also scalable and adaptable to different waste liquors from the pulp and paper industry.

## 1. Introduction

Lignin is a phenolic macromolecule that combines with cellulose to form lignocellulose, the most abundant organic polymer on earth, representing ~30% of the total organic carbon in the biosphere [2]. Lignin accumulates naturally in plant cell walls, a process known as lignification. During pulp production, lignin must be separated from cellulose because it causes paper products to turn yellow over time [3]. Lignin can be separated from cellulose by applying mechanical force, or by chemical modification to make the lignin more soluble, and two major chemical methods have been described: the kraft process and the sulfite process [4]. The waste material from these processes (black liquor and spent sulfite liquor, respectively) is a complex mixture of organic and inorganic molecules. The global paper industry produces more than 5 × 10^7^ tonnes of modified lignin every year, dissolved in the waste liquor [5]. Approximately 99% of this waste liquor is incinerated to produce energy, and the remainder is used to produce low-value products such as wood glue and wetting agents. However, lignin is a versatile raw material that can be used to manufacture many valuable products, including vanillin, vanillic acid, synthetic tannins and polymer filters [6,7].

## 2. Characteristics of Lignin Solutions

### 2.1. Black Liquor

Black liquor is a highly alkaline (pH 13–14) [8] and viscous liquid by-product of the kraft process. The process was invented in 1879 by the German chemist Carl Ferdinand Dahl in Gdansk [9]. The high lignin content (kraft lignin) gives the black liquor its characteristic dark brown to black color [10]. Black liquor is rich in organic molecules and inorganic pulping chemicals [11]. The exact composition varies according to the pulping method and the properties of the wood. Typical black liquor components are listed in Table 1. The average molecular mass (M_w_) of kraft lignin varies between 1100 and 6500 g∙moL^−1^, which is lower than soda-anthraquinone lignin, organosolv lignin and ethanol process lignin. The polydispersity (M_w_/M_n_) ranges from 1.8 to 3.6 [12,13,14,15,16,17]. Furthermore, elementary analysis shows that lignin from black liquor has a higher carbon content than the other types of lignin listed above, probably due to dehydration during the pulping process [12]. An important property that determines the reactivity (Mannich reactivity) of lignin is the number of phenolic hydroxyl groups. Kraft lignin and soda-anthraquinone lignin have the highest ratio of phenolic hydroxyl groups among the types of lignin listed above [12].

### 2.2. Spent Sulfite Liquors

Spent sulfite liquor is a highly acidic (pH ~ 2) liquid by-product of the sulfite process. Like black liquor, spent sulfite liquor has a dark brown color, as well as a high content of organic molecules and inorganic pulping chemicals such as sulfurous acid and alkali salts [9]. It also contains large quantities of various monosaccharides derived from degraded hemicellulose, although the precise composition depends on the wood source. Typical spent sulfite liquor components are listed in Table 2. Spent sulfite liquor is generally burned for energy production (the calorific value of lignin is 26,255 kJ∙kg^−1^ compared to 41,840 kJ∙kg^−1^ for oil [18]) and the pulping chemicals are regenerated in a concentration step [19].

Relatively little is known about the molecular weight distribution of lignosulfonate molecules in spent sulfite liquor. This has been investigated by size-exclusion chromatography when the raw material for the liquor was beech wood, revealing an average molecular mass (M_w_) of 5700 g∙moL^−1^ and a polydispersity (M_w_/M_n_) of 5.8 [26]. Another study revealed that the molecular weight distribution of lignosulfonate in 29 spent sulfite liquor samples was 1030 g∙moL^−1^ and did not depend on the pulping conditions [27].

## 3. Technologies for Lignin Purification

**Figure 1:**
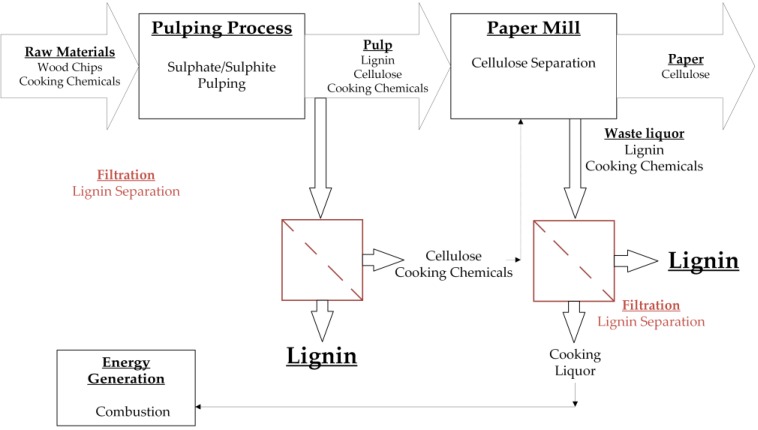
Summary of different strategies in which membrane fractionation can be used for the treatment of waste liquor from the pulp and paper industry. During the pulping process, the raw material is separated into cellulose and lignin. The pulp can be treated by membrane filtration to isolate lignin molecules. The remaining solution can be recycled to the paper production. Additionally, the waste liquor from the paper mill can be treated by membrane filtration to isolate the containing lignin. The remaining solution can be recycled for energy generation.

The waste liquor from the pulp industry is contaminated with pulping chemicals such as sodium hydroxide and sodium sulfide [6]. There are currently no cost-efficient purification strategies, so only a small quantity of valuable lignin products are manufactured. Several methods have been developed to recover lignin from solution, and the current, state-of-the-art solution involves a combination of precipitation and membrane filtration, such as microfiltration (MF), ultrafiltration (UF) and nanofiltration (NF) [28,29]. In the first step, the solubility of lignin is reduced by adding chemicals such as carbon dioxide, sulfuric acid, or chlorine dioxide, and the precipitated lignin is then separated by membrane filtration. Flux decline is one of the greatest challenges during filtration [6] and is difficult to prevent because of the wide molecular weight distribution of the lignin molecules (0.1–400 kDa) [15,26]. Membrane technology is a promising method to achieve efficient lignin recovery, and numerous studies have been carried out to investigate the ability of membranes to recover lignin precipitated from solution (Table 3). Current approaches for the recovery of lignin using membranes are summarized in Figure 1. Ceramic membranes in particular are ideal for the treatment of spent sulfite and black liquors because of their ability to tolerate extreme pH values.

### 3.1. Membrane Technology for the Treatment of Black Liquor

One of the first examples of lignin recovery by membrane filtration was published in 1988 [18]. The authors investigated the chemical reactivity of lignin as a co-polymer during the production of phenolic plastics, as well as the separation of high-molecular-weight lignin using a 10 kDa polysulfone membrane. They were able to increase the lignin concentration in the retentate by ~174% (from 60.69 to 166.4 g∙L^−1^) by applying a permeate/concentrate ratio of four resulting in a lignin purity of 63% in the concentrate and 17% in the permeate. After membrane filtration, they precipitated the lignin fraction with sulfuric acid at pH 2. The purified lignin performed better as a co-polymer for the production of plastics than chemically-modified lignin.

Later studies compared the diafiltration and acid precipitation of black liquor [30,31]. Diafiltration was achieved using a plate-and-frame module and 0.36 m^2^ polymer membranes with molecular weight cut-off (MWCO) values ranging from 6 to 50 kDa. The black liquor was maintained at 60 °C during the diafiltration step. The authors separated 90% of all membrane-permeable components of the liquor with a liquor/water ratio of 2:3. They achieved an average membrane flux of 90 L∙m^−2^∙h^−1^ with the 25 kDa membrane and recovered 54% of the initial lignin concentration, whereas acid precipitation achieved a lignin recovery of 95%. The diafiltration of black liquor has also been tested using polysulfone and polyethersulfone UF membranes with MWCO values of 4, 8 and 20 kDa [8]. Filtration was carried out at a temperature of 60 °C, a cross-flow velocity of 4 m∙s^−1^, and a transmembrane pressure (TMP) of 1.0–7.0 bar. The authors retained 45% of the lignin using the 20 kDa membrane, 67% using the 8 kDa membrane and 80% using the 4 kDa membrane. With a feed/water ratio of 1:1 they achieved a lignin purity of 78%, but the flux declined by 94%. They concluded that membranes with a higher MWCO increase the purity of the recovered lignin, whereas membranes with a low MWCO are better if the aim is to achieve a high lignin concentration.

A further study compared the ability of UF and selective precipitation to complete the fractionation of the lignin resulting from the black liquor of the pulping process [46]. For the UF, tubular ceramic membranes with a MWCO value of 5, 10, and 15 kDa were investigated, and the lignin was fractionated by successively increasing the MWCO values. The selective precipitation was carried out with different concentrations of sulfuric acid. Analytical results obtained with size exclusion chromatography showed that precipitation as well as UF with ceramic membranes are effective techniques to fractionate lignin in black liquor. For the UF, the polydispersity and the average molar mass of the lignin fractions decrease with the MWCO value of the ceramic membrane. Fourier transform infrared spectroscopy, thermal analysis, and proton nuclear magnetic resonance spectroscopy (H-NMR) showed that lignin from UF contains less contamination with hemicellulose as precipitated lignin fractions. Additionally, the H-NMR spectra showed that UF slightly depolymerized lignin.

Padilla et al. [47] investigated the ultrafiltrated lignin fractions obtained from industrial black liquor after alkaline digestion of tropical woods as stabilizers of liquid-liquid and solid-liquid interfaces. The UF was carried out with diluted black liquor (15% solid content). The diluted black liquor was prefiltered with a 100 kDa polysulfone membrane. The permeate of this filtration step was used as a feed for the next filtration process with the 30 kDa polysulfone membrane. This procedure was repeated for a 10 kDa polysulfone and a 5 kDa and 1 kDa cellulose acetate membrane. It was observed that an increase of the molecular weight of the lignin fractions resulted in a higher surface activity. Smaller lignin molecules have an unbalanced ratio of hydrophobic and hydrophilic groups. Thereby the hydrophilic becomes more dominant and the surface activity is reduced. It follows from the foregoing that the high molecular weight lignin fraction has the highest dispersion abilities. This was demonstrated by viscosity measurements of bentonite mixed with high molecular lignin. Furthermore, it has been shown that only the high molecular weight fraction of lignin has a measurable impact on the viscosity of a bentonite-lignin solution.

A further study also investigated the behavior of fractionated and ultrafiltrated black liquor relating to the glass transition temperature and the chemical composition. The cross flow ultrafiltration was carried out with aluminum oxide, titanium oxide, and regenerated cellulose membranes with MWCO values of 1, 5, and 10 kDa. The feed had a temperature of 40–65 °C during the ultrafiltration and the TMP was ~3.5 bar. Overall, six different lignin fractions have been isolated by membrane filtration (0–1 kDa, 0–5 kDa, 1–5 kDa, 5–10 kDa, >5 kDa, >10 kDa). In the first step, the black liquor was filtered with the 5 kDa membrane. The permeate of this filtration was additionally fractionated with the 1 kDa membrane. The retentate of the first step was fractionated with a 10 kD membrane. Retentate and permeate of both filtration steps were stored. The glass transition temperature was strongly dependent on the molecular weight of the lignin fraction and varied from 70 to 170 °C with a rising molecular weight from the 0–1 kDa fraction to the >10 kDa fraction. This relation has been demonstrated earlier by solvent fractionated lignin. The increasing molecular weight of the lignin resulted in an increasing entanglement and rigidity of the lignin molecule. That leads, finally, to an increase in the glass transition temperature. The analysis of the chemical structure of the lignin fraction was carried out by phosphor nuclear magnetic resonance spectroscopy (P-NMR). It was shown that the number of phenolic hydroxyl groups decrease whereas the number of aliphatic hydroxyl groups increase when the molecular size of the lignin fraction increased. The carboxylic groups were not affected by the molecular weight of the black liquor fraction. This behavior is in accordance with the mechanism of lignin degradation during the cocking process. In summary, this study showed that UF of black liquor allows the selective extraction of lignin fractions with particular termo-mechanical properties [48].

Dead-end and cross-flow filtration using various cellulose acetate UF and NF membranes have been tested for the treatment of black liquor, to investigate the impact of process and membrane parameters such as the MWCO (1, 5, and 10 kDa), Reynolds number (3–20,000), TMP (2.75–8.27 bar) and feed concentration [32]. The authors found that a higher MWCO increases the flux but reduces the lignin retention efficiency, and that there was a relationship between the Reynolds number and the flux. The optimal Reynolds number was 17,500 for the radial stirred cell dead-end system, 1200 for the rectangular stirred cell dead-end system, and four for the radial cross-flow system. A higher Reynolds number resulted in slower surface layer formation and facilitated backward diffusion. This confirmed that convection transport is the predominant membrane transport mechanism at high Reynolds numbers based on the low osmotic pressure, resulting in a higher membrane flux. The opposite effect was caused by irreversible membrane fouling, leading to a slower flux increase at high Reynolds numbers and, finally, to an asymptotic approximation of a maximum permeate flux. The authors found that lignin retention increased with higher Reynolds numbers and ran asymptotically to a maximum value. Furthermore, they recognized that concentration polarization increases at high pressures and feed concentrations, in agreement with other studies [30,31,36]. The flux decline during filtration was, therefore, shown to be controlled by the osmotic pressure during cross-flow filtration and by surface layer formation during dead-end filtration.

The ultrafiltration of black liquor has also been carried out using a 15 kDa aluminum oxide/titanium oxide membrane with seven channels (diameter = 6 mm) [33]. The lignin retention was 30%–40% depending on the feed temperature (60 °C, 75 °C, or 90 °C). The crossflow velocity was kept constant at 4.5 m∙s^−1^, causing a pressure drop of 0.9 bar. The retention declined at higher temperatures because the lignin became more soluble, but the retention of the inorganic components varied depending on their valency. The retention of monovalent ions such as sodium and potassium was nearly zero, whereas multivalent ions such as iron, calcium, magnesium, and manganese showed retention values of 50%–90%, probably due to the association between multivalent ions and organic material. The lignin retention at 90 °C was 90%, increasing the concentration from 56 to 160 g∙L^−1^. Similar results were achieved in later studies [34,35,54].

Black liquor taken directly from the pulping process has been separated at temperatures exceeding 100 °C using ceramic UF membranes with MWCO values of 5 and 15 kDa [37]. Lignin retention declined from 45% at 90 °C to 22% at 145 °C for the 5 kDa membrane with a TMP of 1 bar. This study indicated that the maximum rate of temperature change should be 2–3 °C∙min^−1^ in order to prevent membrane bursting [37]. Black liquor has also been separated using ceramic UF and NF membranes with MWCO values of 1, 5, and 15 kDa to extract the small lignin fraction in a highly pure form [38]. The authors reported that black liquors from hardwood and softwood showed different flux behaviors, with the hardwood liquor achieving a lower flux due to the higher average molecular weight of the lignin fraction. After precipitation, the lignin fraction with a molecular weight less than 1 kDa increased from 19% to 44%.

The fractionation of black liquor has also been compared using a variety of membranes made from different materials, e.g., cellulose acetate, aluminum oxide, polyaryl ether ketone, polyacrylonitrile, and polyethersulfone. The percentage of each lignin fraction is summarized in Table 4. Continuous filtration was conducted for 40 days using a 0.2 µm ceramic membrane at a TMP of 2 bar and a cross-flow velocity of 2.3 m∙s^−1^. The flux declined from 310 to 150 L∙m^−2^∙h^−1^ over 16 days, and the lignin retention was 80% [39]. The filtration of black liquor with zirconium dioxide membranes achieved a pressure‑independent flux of 52 L∙m^−2^∙h^−1^ over the pressure range 3–5 bar at a cross-flow velocity of 2.1 m∙s^−1^. The authors proposed that the lignin absorbed specifically at the membrane surface and formed a gel, as confirmed by the retention of organic molecules caused by the compression of the gel layer [40].

The filtration of black liquor with a rotating disk membrane module using a 5 kDa cellulose acetate membrane required a centrifugation step due to the large number of fibers [41]. The study considered the effect of the disk rotation speed (0–600 rpm), TMP (~3–8 bar) and stirring speed (0–1000 rpm). An increase in the disk rotation speed from 0 to 450 rpm increased the flux by 23%, and the flux increased by 60% after filtration for 60 min compared to a non-stirred system. The rotating membrane induced turbulence on the feed side which reduced fouling. A 33% higher flux was achieved by increasing the feed stirring speed from 0 to 1000 rpm [41].

More than 90% of organic contaminants were removed from black liquor using a cascade of ultrafiltration, acid precipitation, crystallization and nanofiltration (0.4 kDa), increasing the concentration of lignin in the retentate from 82 to 230 g∙L^−1^ [42]. Another combination of ultrafiltration and nanofiltration was shown to be economically viable for the production of a pure lignin fraction [44]. The UF step comprised a 20 kDa aluminum oxide/titanium oxide membrane at 90 °C with a TMP of 2.0 bar and a cross-flow velocity of 5 m∙s^−1^, whereas the NF step comprised a 1 kDa aluminum oxide membrane, increasing the flux by 48%. The lignin concentration increased from 62 to 285 g∙L^−1^. Pretreatment of the black liquor increased the economic value of the purified lignin by 450%, although the use of ceramic membranes increased the process cost by 300% [44].

Remarkable differences, related to the solubility and the viscosity, were observed by comparison of lignin obtained from UF (5 kDa) and lignin from the Lignoboost process. The filtration method was a large scale version of the previously described system from Keyoumu et al. [38]. First of all, both lignin types were soluble in moderately polar solvents (ethanol, acetic acid, methanol). However, highly polar solvents like water and apolar solvents like hexane are quite poor solvents for lignin. Nonetheless, the low molecular lignin from the UF was easier to dissolve in good lignin solvents than the unfractionated lignin from the Lignoboost process. Additionally, the viscosity of the filtered lignin was lower at the same concentration than lignin obtained from the Lignoboost process [45].

### 3.2. Membrane Technology for Treatment of Spent Sulfite Liquors

In contrast to the filtration of black liquor, relatively little progress has been made with the development of filtration strategies for spent sulfite liquor. The recovery of lignosulfonates using polysulfone, cellulose acetate, and fluoropolymer UF and NF membranes with MWCO values of 1, 5, 10, 20, 50, and 100 kDa has been studied in a stirred filtration cell at different TMPs (1.0–7.6 bar) with three different dilutions of the spent liquor (neat, 1:1 dilution, and 1:5 dilution) [49]. As anticipated, the flux increased at higher MWCO values and a wider size range of lignosulfonates passed through the fluoropolymer membranes because the MWCO range was broader. Therefore, there was greater flux through the fluoropolymer membranes but less retention compared to the polysulfone membranes. The dilution experiments showed that the lignin rejection efficiency declined but the permeate flux increased at higher dilutions. The membrane pores, blocked by low-molecular-weight lignin, were continuously cleaned, hence the lower rejection, but rejection increased in line with the TMP due to the effects of concentration polarization. Polysulfone membranes with high MWCO values are therefore ideal for the recovery of lignosulfonates because of their high flux and >80% rejection [49].

Lignosulfonate fractionation by amine extraction and ultrafiltration has been compared using polyethersulfone and cellulose UF membranes in cross-flow mode with MWCO values of 1, 10, 50, and 100 kDa. Amine extraction produced three fractions at a time and consumed large amounts of organic solvent, whereas the UF membranes generated five fractions and thus reduced the polydispersity within each fraction. Ultrafiltration also provided more information about the mass distribution in the spent sulfite liquor components. However, there was no significant difference between the methods in terms of total lignin recovery [50].

Tubular ceramic aluminum oxide/titanium oxide membranes have been tested for the treatment of spent sulfite liquor to reduce the chemical oxygen demand as well as residual lignin levels. MF, UF and NF membranes with pore sizes from 0.05 to 0.2 µm and MWCO values ranging from 1 kDa to 20 kDa were tested in different single and multiplex configurations to identify the most efficient strategy [51]. The authors concluded that a two-stage MF/UF process achieved the most promising results, reducing the chemical oxygen demand by 45% and the concentration of the residual lignin by 73% [51]. The ideal configuration comprised a 0.1-µm MF membrane with a crossflow velocity of 5.6 m∙s^−1^ and a 20 kDa UF membrane with a crossflow velocity of 4.0 m∙s^−1^ with an overall TMP of 2 bar.

Flat cellulose acetate, regenerated cellulose, and polysulfone membranes with MWCO values of 0.5, 2, 3, and 10 kDa were used for the separation of hemicellulose and lignosulfonates from spent sulfite liquor. The filtration was carried out in a stirred batch filtration unit under a TMP of 3.6 bar. The flux decreased for the 3 kDa membrane from 39.6 L∙m^−2^∙h^−1^ to 8.4 L∙m^−2^∙h^−1^ during a 180 min batch fermentation. In this time, a volume reduction of 60% was achieved. The filtration results showed that lignosulfonate as well as hemicellulose can be recovered from all tested membranes. After a volume reduction of 65%, a final lignin rejection of 65%, 52%, and 77% could be achieved for the 10, 3, and 2 kDa membrane, respectively. The 3 kDa membrane, made of regenerated cellulose, gave the best results for ligninsulfonate recovery with a high rejection and selectivity of lignosulfonate [52].

Flat UF and NF membranes made of regenerated cellulose have been investigated for the pretreatment of spent sulfite liquor for the bio-based succinic acid production with *Actinobacillus succinogenes* and *Basfia succiniciproducens*. In this study, membranes with MWCO values of 10, 5, 3, and 0.5 kDa were used. The UF experiments were carried out in a stirred ultrafiltration cell at a TMP of 3.0 bar. Per batch, 400 mL of 10 times diluted spent sulfite liquor was filtered. Thereby a flux of 35.7 L∙m^−2^∙h^−1^ was achieved for the 10 kDa. The lignosulfonate concentration in the permeate increased from ~37 g∙L^−1^ to 104.3, 119.2, 157.1 g∙L^−1^ for the 10, 5, and 3 kDa membranes, respectively. The NF with the 0.5 kDa membrane was conducted with a vibratory share-enhanced filtration unit that used oscillatory vibrations to induce high shear rates on the membrane surface whereby membrane fouling was reduced. The seven times diluted spent sulfide liquor had a temperature of 54–75 °C during the filtration. The TMP was 20.8 bar at the beginning of the filtration and was gradually increased to 31 bar at the end of the filtration. The flux decreased from 44.0 L∙m^−2^∙h^−1^ to 10.7 L∙m^−2^∙h^−1^ in the course of the filtration. The lignosulfonate concentration increased from the initial concentration of 60.0 g∙L^−1^ to 166.1 g∙L^−1^ in the retentate. The 0.5 kDa NF membrane achieved a lignosulfonate recovery of 95.6%. The fermentation experiments with the permeate from the NF resulted in a production of ~ 52 g∙L^−1^ succinic acid whereas the use of the permeate from the 3 kDa UF membrane resulted in 71.8 g∙L^−1^ succinic acid [53].

## 4. Membrane Fouling and Cleaning Strategies

Membrane fouling is defined as the irreversible formation of deposits on the active surface of a membrane, causing a decline in flux and a loss of performance. This is a particular problem during the filtration of brackish water, seawater, and industrial wastewater [54]. The filtration of black liquor and spent sulfite liquor is prone to fouling and this becomes a significant cost factor in the context of industrial-scale processes. Therefore, effective cleaning strategies are necessary to reduce the rate of fouling and prolong the life of membranes.

One potential solution involves a combination of rinsing and chemical cleaning [33]. The membrane was rinsed with permeate collected during the filtration because this has the same pH as the original feed solution and potential foulants are, therefore, likely to dissolve. Rinsing was carried out at the operational temperature and at a TMP of 0.5 bar. Chemical cleaning was carried out with 0.25 wt % Ultrasil 11 (Henkel, Germany) at 60 °C and a TMP of 0.5 bar until the initial pure water flux was restored. Ultrasil 11 is an alkaline cleaning agent free of ethylenediaminetetraacetic acid (EDTA) and nitrilotriacetic acid (NTA). The initial pure water flux of the ceramic membrane (MWCO = 15 kDa) was completely recovered [33]. Rinsing alone was shown to restore 70%–80% of the initial pure water flux, whereas the combination of rinsing and chemical cleaning restored 90%–95% of the initial value [8].

A four-step process has been developed for inorganic membranes: 30 min washing with tap water, 30 min washing with 0.2 mol∙L^−1^ sodium hydroxide, 30 min in the furnace at 550 °C, and 30 min washing with 0.2 mol∙L^−1^ hydrochloric acid. The initial pure water flux was completely recovered [39]. For organic membranes, the same procedure can be used without the furnace step, restoring 50%–80% of the initial pure water flux [39].

**Figure 2:**
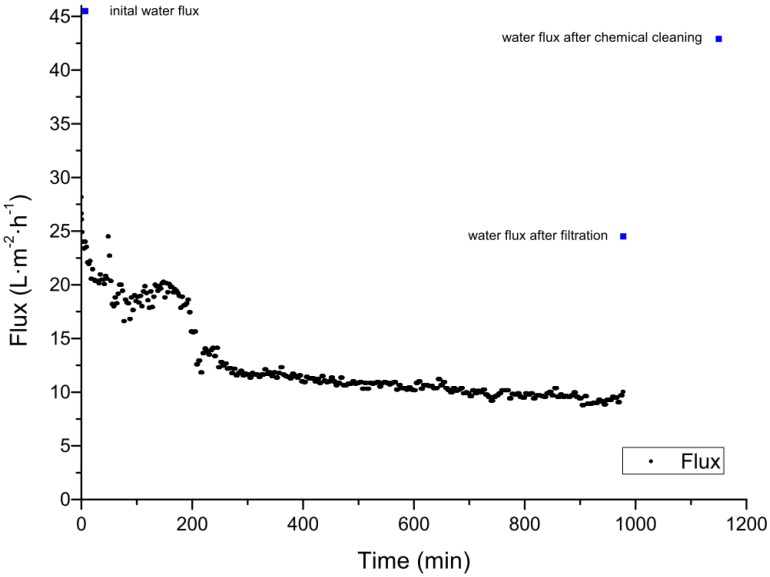
Filtration of spent sulfite liquor using a 5 kDa ceramic membrane at 60 °C, with an average transmembrane pressure (TMP) of 0.4 bar. The blue dots represent the initial pure water flux, the water flux after the filtration, and the water flux after the chemical cleaning.

The cleaning of ceramic membranes after the filtration of spent sulfite liquor was carried out using 1% sodium hydroxide at 60 °C for 1 h followed by rinsing with distilled water. For a 20 kDa membrane, the pure water flux was restored to 98% of the initial value, and for a 1 kDa membrane cleaning restored 80% of the pure water flux [51]. A back-flush for 10 s at a pressure of 4 bar was also shown to reduce fouling, but cleaning was more efficient when there was a longer interval between flushes. When the interval was 60 min the average flux increased by 16%, but with an interval of 120 min the average flux by 48% [51]. Our experiments for the treatment of spent sulfite liquor also focused on chemical cleaning strategies for ceramic membranes. Using an aluminum oxide UF membrane with a MWCO value of 5 kDa manufactured by atech innovations (Gladbeck, Germany), the initial clean water flux was completely recovered after cleaning for 4 h with Ultrasil 11 at 60 °C and a TMP of 0.5 bar. The permeate flux over a filtration run lasting 17 h is shown in Figure 2. The permeate flux declined after 3 h by approximately 60% from 28.0 to 11.8 L∙m^−2^∙h^−1^. The average permeate flux was 12.5 L∙m^−2^∙h^−1^ The clean water fluxes before the filtration of spent sulfite liquor, after filtration, and after chemical cleaning are compared in Table 5 and are shown in Figure 2. After the treatment of spent sulfite liquor, the membrane was physically cleaned with pure water, restoring 56% of the initial pure water flux, and chemical cleaning restored the remaining 44%. The flux-time curve of the filtration shows the typical profile, comprising a rapid initial decline (0–50 min) caused by fast pore blocking followed by the formation and growth of a cake layer, adsorption, and concentration polarization (50–1000 min). Steady-state flux, reflecting an equilibrium between membrane and cake layer resistance at the TMP, cross flow velocity, and temperature shown, was not accomplished in this experiment because the filtration was terminated after 1000 h [55]. Irreversible fouling was not observed after the first filtration cycle. Chemical cleaning restored the initial water flux completely, therefore, complex and labor-intensive cleaning strategies to recover the initial flux was not necessary. 

## 5. Summary

Every pulping process in the world produces vast amounts of wastewater containing modified lignin, which is usually incinerated to provide energy despite the potential to recover the lignin and use it for the manufacture of valuable chemical products. Membrane technology offers an economical and environmentally beneficial platform for the recovery of lignin. Organic and inorganic membranes with MWCO values of 0.4–100 kDa have been investigated, using diverse methods such as radial, rectangular cross-flow, and dead-end filtration combined with stirred rotating disc modules and plate-and-frame modules. The impact of different filtration parameters such as TMP, feed temperature, cross-flow velocity, and the Reynolds number have been studied in detail, resulting in processes that achieve lignin concentrations of up to 285 g∙L^−1^ and purities of up to 78% [8,44]. Membrane filtration combined with diafiltration also reduced the ash content of kraft liquor from 4.7% to 2.7% [8].

Membrane cleaning strategies have been developed to prevent fouling and restore membrane flux after lignin purification. A combination of rinsing (with permeate) and cleaning with Ultrasil 11 achieved the best results, and regular back-flushing was shown to prevent fouling and increase the average flux by up to 48% [51].

Economic models suggest that membrane-based recovery can produce pure lignin at a cost of €46–120 per tonne, although the inclusion of a diafiltration step would cost more [43]. Therefore, the membrane-based filtration of wastewater from the pulp and paper industry holds great promise for the economical and environmentally-sustainable recovery of concentrated and highly pure lignin, with strategies in place to maximize membrane performance and longevity. Membrane technology could face strong competition from the Lignoboost process, but the latter is only suitable for black liquor and is also more difficult to scale-up. Therefore, membrane technology offers a unique combination of versatility, scalability, and economy that makes it suitable for the recovery of lignin from diverse industrial processes.

## Figures and Tables

**Table 1:** Typical composition of black liquor [8].

Component	Variation Range
dry weight	(12–18) wt %
polyaromatic components	(30–45) wt %
saccharinic acid	(25–35) wt %
formic acid	(0–10) wt %
acetic acid	(0–10) wt %
extractives	(3–5) wt %
methanol	1 wt %
inorganic elements (mainly sodium)	(17–20) wt %
lignin	(45–65) g∙L^−1^

**Table 2:** Typical composition of spent sulfite liquors before evaporation [19,20,21,22,23,24,25].

Component	Variation Range
dry weight	(128–220) g∙L^−1^
acetic acid	(4.7–9.3) g∙L^−1^
extractives	~1 g∙L^−1^
lignosulfonate	(59–120) g∙L^−1^
pH value	(1.7–3.4) g∙L^−1^
arabinose	(1.0–7.8) g∙L^−1^
xylose	(0.8–26.7) g∙L^−1^
mannose	(4.0–16.16) g∙L^−1^
galactose	(0.2–5.34) g∙L^−1^
glucose	(1.7–3.28) g∙L^−1^
fucose	0.4 g∙L^−1^
rhamnose	~1 g∙L^−1^
furfural	(0.03–2.00) g∙L^−1^
hydroxymethylfurfural	~0.34 g∙L^−1^
ash	(19.8–20.8) g∙L^−1^
density	(1180–1050) g∙L^−1^
methanol	<1 g∙L^−1^

**Table 3:** Overview of recent studies investigating membrane technologies for the treatment of black liquor and spent sulfite liquor from pulp and paper mills.

Process	Source	Membrane Type	Membrane Material	Reference
UF	black liquor	–	polymer	[18]
UF	black liquor	flat membranes	polymer	[30,31]
UF	black liquor	tubular membranes	polymer	[8]
UF	black liquor	flat membranes	polymer	[32]
UF	black liquor	tubular membranes	ceramic	[33,34,35]
UF	black liquor	flat membranes	polymer	[36]
UF	black liquor	tubular membranes	ceramic	[37]
NF/UF	black liquor	tubular membranes	ceramic	[38]
MF/UF	black liquor	tubular membranes/flat membranes	polymer/ceramic	[39]
UF/NF	black liquor	tubular membranes	ceramic	[40]
UF	black liquor	flat membranes	polymer	[41]
UF/NF	black liquor	flat membranes	polymer	[42]
–	black liquor	flat membranes	–	[43]
UF/NF	black liquor	tubular membranes	ceramic	[44]
UF	black liquor	tubular membranes	ceramic	[45]
UF	black liquor	tubular membranes	ceramic	[46]
UF	black liquor	flat membranes	polymer	[47]
UF	black liquor	tubular membranes	polymer/ceramic	[48]
UF	spent sulfite liquor	flat membranes	polymer	[49]
UF	spent sulfite liquor	flat membranes	polymer	[50]
MF/UF/NF	spent sulfite liquor	tubular membranes	ceramic	[51]
UF	spent sulfite liquor	flat membranes	polymer	[52]
UF/NF	spent sulfite liquor	flat membranes	polymer	[53]

**Table 4:** Molecular weight distribution of lignin in black liquor [39].

Molecular Weight in kDa	Percentage Abundance
>60	61.8
60–30	21.8
30–10	1.2
10–6	1.8
6–3	2.4
<3	1.0

**Table 5:** Water fluxes at different TMPs before the filtration of spent sulfite liquor, after filtration, and after chemical cleaning.

Pressure (bar)	Water Flux before Filtration (L∙m^−2^∙h^−1^)	Water Flux after Filtration before Chemical Cleaning (L∙m^−2^∙h^−1^)	Water Flux after Chemical Cleaning (L∙m^−2^∙h^−1^)
0.5	45.5	24.5	42.9
1.0	88.7	51.4	94.1
1.5	140.7	79.0	145.3
2.0	197.5	110.6	193.0
